# Dietary Sodium Butyrate Alleviates Thiram-Induced Tibial Dyschondroplasia in Broilers by Improving Redox Homeostasis and Upregulating the Nrf2/HO-1 Pathway

**DOI:** 10.3390/antiox15101302

**Published:** 2026-10-09

**Authors:** Jiawei Wang, Yongbo Hao, Shaoyang Han, Feifei Luo, Ziyi Zheng, Shuo Sun, Xi Sun, Yongkang Diao, Jiahao Zhao, Suqiao Chu, Lijun Hou, Boyang Shi, Hui Chen, Chong Li

**Affiliations:** 1 College of Animal Science and Technology, Hebei Agricultural University, Baoding 071000, China; wjwyhzfl@163.com (J.W.); 13463360103@163.com (Y.H.); 13703108685@163.com (S.H.); www816823@163.com (Z.Z.); 13349484274@163.com (S.S.); 18131965911@163.com (X.S.); dyk0509@163.com (Y.D.); 17332157739@163.com (J.Z.); 2 Bureau of Agriculture and Rural Affairs of Jinzhou City, Jinzhou 052200, China; zdm920530@126.com; 3 Shijiazhuang Animal Husbandry Technology Extension Station, Shijiazhuang 050000, China; chusuqiao@163.com; 4 Bureau of Agriculture and Rural Affairs of Langfang City, Langfang 065000, China; 13303361199@163.com; 5 Hebei Provincial Station of Veterinary Drug and Feed, Shijiazhuang 050035, China; superayangyang@163.com; 6 Hebei Technology Innovation Center of Forage Resource Development and Efficient Utilization, Baoding 071000, China

**Keywords:** tibial dyschondroplasia, sodium butyrate, oxidative stress, gut microbiota, bone metabolism

## Abstract

Oxidative stress-induced impairment of chondrocyte differentiation and bone homeostasis is pivotal in tibial dyschondroplasia (TD). Sodium butyrate has been shown to modulate gut microbiota, improve redox homeostasis, and support skeletal metabolism; however, whether it exerts protection against TD specifically through oxidative stress modulation remains unclear. A total of 210 one-day-old male Arbor Acres broilers were randomly allocated to three treatment groups, with seven replicate pens per group and 10 birds per pen. The birds were housed in a windowless, climate-controlled indoor poultry house with floor pens and rice-husk bedding. In a thiram-induced broiler TD model, dietary sodium butyrate (500 mg/kg) reshaped the gut microbiota by increasing beneficial genera (*Ligilactobacillus*, *Lactobacillus*, *Blautia*, and *Mediterraneibacter*), decreasing *Escherichia-Shigella* abundance, and elevating ileal butyrate concentration. These shifts co-occurred with mitigated oxidative damage, including reduced ROS and MDA in both serum and ileal mucosa, partial elevations in SOD and GSH-Px activities, upregulated *Nrf2* and *HO-1* expression in both the ileal mucosa and tibial growth plate, and improved mitochondrial function in the ileal mucosa, as indicated by restored mitochondrial membrane potential, reduced superoxide anion levels, and increased respiratory chain complex I activity. Concomitantly, sodium butyrate improved growth performance and skeletal phenotypes, attenuating growth plate lesions and restoring tibial morphology and mineralization. It also favorably modulated bone metabolism by elevating the OPG/RANKL ratio and upregulating *COL2A1* expression. Correlation analyses linked microbial alterations to antioxidant status and skeletal parameters. Collectively, these findings suggest that sodium butyrate alleviates thiram-induced TD in association with gut microbiota remodeling, which is correlated with enhanced redox homeostasis and bone metabolism, supporting a potential gut–bone axis mechanism.

## 1. Introduction

Oxidative stress has been increasingly recognized as a critical pathogenic driver of skeletal disorders in rapidly growing broilers, disrupting redox homeostasis in growth plate chondrocytes and impairing the coordinated processes of differentiation, vascular invasion, and matrix mineralization essential for normal bone development [1,2]. Tibial dyschondroplasia (TD), characterized by impaired chondrocyte differentiation and reduced vascular invasion in the tibial growth plate [3,4,5], exemplifies such oxidative stress-mediated pathology, leading to lameness, poor growth, and substantial economic losses [6]. Excessive reactive oxygen species (ROS) can trigger chondrocyte apoptosis, disturb redox balance, and limit vascular invasion, thereby worsening cartilage mineralization defects [7]. As a dithiocarbamate fungicide, thiram (tetramethylthiuram disulfide) is commonly applied in agricultural settings for the control of fungal pathogens in crops [8]. Residues from treated plants may persist and subsequently contaminate feed ingredients, posing a risk to poultry [9]. In avian species, exposure to thiram is recognized to trigger tibial dyschondroplasia by impairing normal vascularization and chondrocyte maturation within the growth plate [10]. Consequently, dietary thiram administration has become a standard experimental approach for inducing TD in broiler models [11]. The Nrf2/HO-1 axis serves as a key regulator of cellular antioxidant defenses and is essential for counteracting oxidative injury in various tissues [12,13]. In TD models, impaired *Nrf2* activation and reduced *HO-1* expression are associated with increased chondrocyte apoptosis and growth plate abnormalities [2], underscoring the protective role of this pathway against oxidative stress in poultry skeletal development. However, the underlying mechanisms by which the Nrf2/HO-1 pathway contributes to TD pathogenesis are not yet fully understood. Therefore, elucidating these redox-dependent signaling cascades and developing targeted nutritional interventions are of practical importance.

Bone metabolic homeostasis is contingent upon the balance between osteogenesis and resorption, and the RANKL/OPG system is involved in osteoclast differentiation and bone remodeling [14,15]. RANKL promotes osteoclast formation and activation, whereas osteoprotegerin (OPG) competes with RANK for RANKL, thus curtailing bone resorption; therefore, the OPG/RANKL ratio is often used to assess bone metabolic balance [16,17]. Existing research has demonstrated that oxidative stress may disturb the homeostatic balance of bone metabolism by modulating the expression profiles of *OPG* and *RANKL* [18,19]. This oxidative stress-driven reduction in the OPG/RANKL ratio may enhance osteoclast-mediated bone resorption, which, in the TD-affected growth plate, could perturb the normal progression of endochondral ossification and lead to structural abnormalities [20]. However, the relationship between oxidative stress-associated OPG/RANKL imbalance and TD development remains unclear.

Gut microbial populations and their metabolites, chiefly short-chain fatty acids (SCFAs), are known to regulate host redox balance and skeletal health [21]. SCFAs influence bone metabolism both directly, through effects on osteoclast and osteoblast activity, and indirectly, by modulating intestinal barrier function, systemic inflammation, and oxidative stress [22,23]. Butyrate, a major microbial SCFA, fuels intestinal epithelial cells and has anti-inflammatory, antioxidant, and immunoregulatory effects [24,25]. In poultry, sodium butyrate has been reported to confer multiple health benefits. It strengthens intestinal barrier integrity through the upregulation of tight junction proteins [26,27], favorably reshapes the gut microbial community by enriching beneficial taxa such as Lactobacillus and Blautia, and enhances systemic antioxidant defenses via Nrf2-mediated signaling [28,29]. Moreover, butyrate contributes to skeletal homeostasis by facilitating osteoblast differentiation while restraining osteoclastogenesis through the OPG/RANKL axis [30]. Collectively, these observations support the exploration of sodium butyrate as a potential nutritional strategy for TD intervention. Therefore, we hypothesized that dietary sodium butyrate could alleviate thiram-induced TD in broilers by improving intestinal microbial homeostasis and enhancing antioxidant defense, thereby contributing to the restoration of growth plate redox balance and bone metabolic homeostasis. The purpose of this research was to determine whether dietary sodium butyrate could alleviate thiram-induced tibial dyschondroplasia in broilers and to characterize the potential involvement of the gut–bone axis, intestinal microbiota, mitochondrial function, and the Nrf2/HO-1 antioxidant pathway. This study extends current knowledge by linking sodium butyrate-induced gut microbiota remodeling with intestinal mitochondrial redox status, Nrf2/HO-1 antioxidant signaling, and tibial bone metabolism in a thiram-induced TD model. From a poultry production perspective, these findings suggest that sodium butyrate may represent a potential feed-based strategy for supporting skeletal health and reducing TD-associated growth impairment and lameness. Nevertheless, further studies under commercial production conditions and with different supplementation levels are needed to confirm its practical applicability. To test this hypothesis, we evaluated growth performance, tibial development, oxidative stress markers, Nrf2/HO-1 and OPG/RANKL-related expression, as well as ileal SCFA concentrations and microbial composition.

## 2. Materials and Methods

### 2.1. Chemicals, Experimental Animals, and Design

Tetramethylthiuram disulfide (thiram; Cat. No. M03718066; ≥98% purity) was supplied by Macklin Biochemical Co., Ltd. (Shanghai, China). Sodium butyrate (98%; Cat. No. S30223) was acquired from Shanghai Yuanye Bio-Technology Co., Ltd. (Shanghai, China).

A total of 210 one-day-old male Arbor Acres broilers (mean body weight, 40.8 g; SD, 1.25 g) were used in this experiment. The chicks were procured from Hebei Jiuxing Agriculture and Animal Husbandry Development Co., Ltd. (Xingtai, China). Broilers in the different treatment groups were reared in separate climate-controlled pens. The poultry house was a windowless closed building without outdoor access. A total of 21 floor pens were used (7 replicates per treatment group). Each pen had an area of 1.8 m^2^. Rice husk was used as the bedding substrate. Lighting was provided by LED white-light lamps with a light intensity of 20 lx. During the whole trial phase, all broilers were allowed free and continuous access to the basal diet and clean drinking water, and were reared under a lighting regime consisting of 23 h of illumination and 1 h of darkness. During the first week, the house temperature was maintained at 33 °C; thereafter, temperature was gradually decreased by 2–3 °C per week and stabilized at 28 ± 1 °C starting from day 14. Adequate ventilation was ensured throughout the experiment, and the health status of the broilers was monitored regularly.

After arrival, all experimental broilers were allocated at random into three treatment groups, with 7 replicates per group and 10 broilers per replicate. At the end of the trial, one broiler from each pen, whose body weight approximated the pen mean, was selected for sample collection; seven biological replicates were obtained per treatment group, giving a total of 21 sampled broilers for biochemical, histological, molecular, microbiota, and bone parameter analyses. The rationale for selecting the bird closest to the pen mean was to minimize inter-individual variation within each pen and ensure that the selected samples were representative of the pen average, thereby reducing noise in downstream biochemical and molecular analyses. The experiment lasted for 21 days (Figure 1). The three experimental groups comprised the following: (1) The CON group (control group), in which broilers were fed a basal diet, and (2) the TD group (model group), which was fed the basal diet plus thiram (100 mg/kg) from days 4 to 7 to induce tibial dyschondroplasia (TD). Thiram was thoroughly mixed into the basal diet at a concentration of 100 mg/kg and administered during the feeding period. The selection of 100 mg/kg and the 4-day exposure period (days 4–7) was based on the established protocol described by Zhang et al. [31], which consistently induces TD in broilers with high reproducibility and minimal mortality. (3) The TSB group (sodium butyrate intervention group) was subjected to the same thiram treatment (100 mg/kg, days 4–7) and simultaneously fed the basal diet supplemented with sodium butyrate (500 mg/kg) throughout the experimental period (days 0–21). The 500 mg/kg concentration represents the actual amount of sodium butyrate (98% purity) supplemented, not butyrate equivalent. Sodium butyrate was incorporated into the basal diet by stepwise mixing: first hand-mixed with a small portion of the feed (1:10), then blended with the remaining feed using a mechanical mixer for 15 min to ensure uniform distribution. Homogeneity was confirmed by collecting and analyzing three samples from different layers of the mixed feed using gas chromatography. The experimental basal diet was designed to satisfy or surpass the nutritional standards proposed by the Chinese agricultural industry standard (NY/T 33-2004) [32]. The dietary components and measured nutrient contents are displayed in Table S1.

### 2.2. Growth Performance Assessment

Pen-level body weight (BW) was recorded on days 7, 14, and 21 using a digital electronic scale (Type/Model: ACS-30; Manufacturer: Kaifeng Group Co., Ltd., Yongkang, China; capacity: 30 kg; precision: 10 g). Feed intake (FI) was recorded per pen for the periods 0–7 d, 7–14 d, and 14–21 d. Growth performance metrics (ADFI, ADG, and FCR) were computed per pen for each phase, with the pen serving as the experimental unit.

### 2.3. Clinical Observation and Tibial Lesion Scoring

At 21 days of the trial, broilers in each treatment group were observed for signs of joint swelling and standing difficulties. All broilers were then euthanized, and their tibiae were examined for the presence of typical TD lesions such as white cartilage plugs. Tibial lesions were assigned scores of 0–4 based on Pines et al.’s criteria [33], and the mean lesion score was calculated for each group.

### 2.4. Measurement of Tibial Morphological Parameters

At day 21, broilers were fasted for 12 h prior to slaughter. Birds were electrically stunned in a waterbath stunner (Manufacturer: Nanjing Aneng Technology Co., Ltd., Nanjing, China; frequency 750 Hz, average current 200 mA per bird, sine wave AC) before exsanguination [34], and bleeding was allowed for 3 min. Broilers were humanely sacrificed, and tibiae were promptly harvested for subsequent analysis. All attached muscle, fat and connective soft tissues were completely removed before fresh-weight recording. Bone length was measured from the condylus lateralis proximalis tibiae to the malleolus lateralis distalis tibiae; tibial width was measured at the mid-diaphysis; growth plate width was measured at the widest point of the proximal tibial growth plate on a tangential section. All measurements were performed using a digital caliper (Model: 500-196-30; Mitutoyo Corporation, Kawasaki, Japan, 0.01 mm precision). The tibial growth plate index was determined as growth plate width divided by tibial length. These measurements were performed according to the methods described by Zhang et al. [35].

### 2.5. Determination of Tibial Mineralization Parameters

The right tibiae of broilers from the same batch were selected from each group, and bone mineral density (BMD, g/cm^2^), DXA-derived bone volume, and bone mineral content (BMC, g) were measured using a dual-energy X-ray absorptiometer (InAlyzer, Medikors, Seongnam, Republic of Korea). We acknowledge that DXA provides areal BMD and an estimated bone volume rather than true three-dimensional bone volume; therefore, the term “bone volume” in this study refers to the DXA-derived parameter. In order to measure the ash content in bone samples, the collected tibiae were dried at a constant temperature of 105 °C for 24 h, ground, placed in porcelain crucibles, and ashed at 550 °C for 24 h. After cooling in a desiccator, samples were weighed to calculate ash percentage. Tibial calcium and phosphorus were quantified by ashing followed by acid dissolution. The ash specimens were subjected to digestion in hydrochloric acid solution, and after dilution to volume, were used for the determination of Ca and P contents. The calcium content present in the samples was determined via atomic absorption spectrophotometry (Model: AA-7000; Shimadzu Corporation, Kyoto, Japan). Phosphorus content was determined by the molybdenum blue colorimetric method according to Zinzadze [36]. Briefly, a series of phosphorus standard solutions at different concentrations were prepared and reacted with ammonium molybdate in acidic medium to form a blue complex. After color development, the absorbance of each standard was measured at 660–700 nm to generate a linear calibration curve. Sample phosphorus concentrations were then determined from the standard curve.

### 2.6. Analysis of Histopathological Morphology

On day 21, the left tibial specimens obtained from broilers were fixed in 4% paraformaldehyde, decalcified using 10% (*w*/*v*) EDTA buffer, and then dehydrated, embedded in paraffin, sectioned, and stained with H&E. The histological structure, chondrocyte arrangement, and vascular invasion in different regions of the growth plate (proliferation zone, hypertrophic zone, and maturation zone) were examined under a light microscope. For histological analysis, three non-adjacent sections (5 µm thick) were examined per bird, and five randomly selected microscopic fields were analyzed per section. Images were captured at 100×, 200×, and 400× magnifications using a light microscope (Olympus BX53; Olympus Corporation, Tokyo, Japan) equipped with a digital camera. Quantitative analysis of chondrocyte count, vascular area, and lacunae density was performed using ImageJ software (version 1.53; National Institutes of Health, Bethesda, MD, USA). All measurements were performed in a blinded manner.

### 2.7. Analysis of Oxidative Stress, Antioxidant Parameters, and Bone Metabolism Markers

Wing-vein blood was taken on day 21, centrifuged (3000× *g*, 15 min), and serum was stored at −20 °C until needed. Ileal mucosa was scraped on ice, rinsed with ice-cold saline to remove residual contents, homogenized in saline (1:9, *w*/*v*), and centrifuged to obtain the supernatant. Serum calcium (Cat. No. A006-2), phosphorus (A020-1), and alkaline phosphatase (ALP; A059-1-1) were measured as bone metabolism markers. The same commercial kits (Nanjing Jiancheng Bioengineering Institute, Nanjing, China) were used to measure ROS (E004-1-1), MDA (A003-1), SOD (A001-1), GSH-Px (A005-1), and T-AOC (A015-2) in both serum and ileal mucosa, following the supplier’s protocols.

### 2.8. Measurement of Ileal Mucosal Mitochondrial Function-Related Parameters

Using the same ileal mucosal supernatant prepared as described above, the levels of superoxide anion (O_2_^•−^), mitochondrial membrane potential (MMP), mitochondrial respiratory chain complex I (MRCC I), and mitochondrial respiratory chain complex III (MRCC III) were determined. The O_2_^•−^ and MMP kits were purchased from Solarbio (Beijing, China; Cat. Nos. BC1295 and CA1310, respectively), the MRCC I kit from Beyotime (Shanghai, China; Cat. No. P0423S), and the MRCC III kit from Nanjing Jiancheng Bioengineering Institute (Cat. No. A089-3-1). All steps strictly followed the manufacturers’ guidelines. Mitochondrial membrane potential (MMP) was evaluated using the JC-1 fluorescent probe (Beyotime, Shanghai, China), and the ratio of red (J-aggregates) to green (monomers) fluorescence was calculated to reflect MMP changes. The activity of mitochondrial respiratory chain complex I (MRCC I) was assessed by monitoring NADH oxidation at 340 nm. Complex III (MRCC III) activity was determined by measuring cytochrome c reduction at 550 nm. Superoxide anion (O_2_^•−^) generation was detected using a chemiluminescence-based assay kit. Each sample was run in triplicate, and values were adjusted to protein concentration using the BCA assay (Beyotime, Shanghai, China).

### 2.9. Analysis of Short-Chain Fatty Acids

The extraction and determination of SCFA in the ileal contents followed Gharib-Naseri et al. [37]. Briefly, ~500 mg of ileal contents (wet weight) was acidified with 25% phosphoric acid (1:1, *v*/*v*) and extracted with diethyl ether (1:2, *v*/*v*) by vortexing for 5 min and centrifugation at 12,000× *g* for 10 min at 4 °C. The organic phase was collected, and extraction was repeated twice. The pooled extracts were concentrated under nitrogen, reconstituted in 1 mL of diethyl ether containing 2-ethylbutyric acid (internal standard), and quantified by external calibration using acetate, propionate, butyrate, isobutyrate, and valerate standards (0.1–100 mmol/L). SCFA concentrations were expressed as μmol/g wet intestinal content, and all samples were analyzed in duplicate.

### 2.10. RNA Extraction and RT-qPCR

On day 21, ileal mucosa and tibial growth plate tissues were collected from broilers, rapidly immersed in liquid nitrogen, and held at −80 °C. The total RNA employed in this study was isolated from homogenized tissue samples, and the content and RNA purity were evaluated using a NanoDrop spectrophotometer (Thermo Scientific, Waltham, MA, USA). An equal quantity of RNA was reverse-transcribed to cDNA with the HiScript III RT SuperMix Kit (Vazyme Biotech Co., Ltd., Nanjing, China). Quantitative real-time PCR was performed employing an SYBR Green detection system (Vazyme, Nanjing, China), and all reaction systems and amplification reactions were prepared strictly in accordance with the kit specifications. The transcript levels of oxidative stress-associated genes including *Nrf2* and *HO-1* in both the ileal mucosa and tibial growth plate, as well as bone metabolism- and osteogenesis-related genes (*OPG*, *RANKL*, *COL2A1*, and *RUNX2*) in the tibial growth plate, were quantified. *GAPDH* served as an internal reference gene to calibrate the expression data, and the relative transcription abundance for target genes was computed by the 2^−ΔΔCt^ method. The specific primer sequences are presented in Supplementary Table S2.

### 2.11. Analysis of the Ileal Microbiota

On day 21, ileal content samples were harvested aseptically and preserved at −80 °C for microbial community analysis. The assay focusing on microbial community structure was accomplished utilizing 16S rRNA gene amplicon sequencing technology, with the entire experimental procedure completed by Shanghai Meiji Biomedical Technology Co., Ltd. based in Shanghai, China. Subsequently, genomic DNA was isolated, the V3–V4 hypervariable segments were subjected to amplification using the primer combination of 338F and 806R, followed by high-throughput sequencing on an Illumina MiSeq platform (2 × 300 bp; Illumina, San Diego, CA, USA). Raw sequencing data were analyzed with the QIIME2 platform (version 2024.2), and the DADA2 (version 1.16) plugin was applied to identify amplicon sequence variants (ASVs) [38,39]. Taxonomic classification was carried out by aligning sequences with the SILVA 138 reference database [40]. Multiple alpha diversity indicators including the Shannon, Simpson, Chao1, and ACE indices were computed, while beta diversity assessments consisting of PCoA based on Bray–Curtis dissimilarity and ANOSIM tests were also implemented. Raw 16S rRNA amplicon sequencing data are stored in NCBI SRA (BioProject: PRJNA1458744).

### 2.12. Statistical Analysis

Microbiome data were processed on the Majorbio platform. Other statistical analyses used SPSS 26.0 (IBM, Armonk, NY, USA). Data normality and homogeneity of variance were assessed by Shapiro–Wilk and Levene’s tests, respectively. Results are expressed as mean ± SEM. Group comparisons were performed using one-way ANOVA with Tukey’s post hoc test when assumptions were met; otherwise, the Kruskal–Wallis test with Dunn’s multiple comparisons was applied. For longitudinal BW and FI data (days 7, 14, 21), a repeated-measures mixed-effects model was used with treatment, time, and their interaction as fixed effects, and pen as random effect, accounting for repeated correlations within pens. One-way ANOVA was applied for non-repeated measures. Statistical significance was set at *p* < 0.05. Figures were created in GraphPad Prism 10 (GraphPad Software, San Diego, CA, USA). For growth performance, pen served as the experimental unit; individual broilers served as the experimental unit for all other measurements unless otherwise indicated. Linear regression was used to assess associations between oxidative stress markers (serum and ileal ROS and MDA) and tibial development parameters (growth plate index and BMD). The coefficient of determination (r^2^) and *p* values were calculated for each regression model. All biochemical, molecular, histological, bone, SCFA, and microbiota analyses were performed with *n* = 7 biological replicates (one bird/pen, 7 pens/group), with growth performance also using *n* = 7 pens/group. Histology analyzed three sections per bird, and microbiota sequencing covered all 21 samples (7/group). Exact *n* values are provided in each figure or table legend.

## 3. Results

### 3.1. Growth Performance

At day 7, BW, ADFI, and ADG did not differ significantly across the three groups (*p* > 0.05). At day 14, the TD and TSB groups showed significantly lower BW, ADFI, and ADG than the CON group (*p* < 0.05); the TSB group demonstrated a marked increase in BW over the TD group (*p* < 0.05), but its ADFI and ADG were not markedly different from those in the TD group (*p* > 0.05). At day 21, BW, ADFI, and ADG differed significantly among all three groups (*p* < 0.05) (Figure 2A–C). At no time point did FCR differ significantly among the three groups (*p* > 0.05) (Figure 2D).

### 3.2. Clinical Manifestations and Pathological Characteristics

Broilers in the CON group exhibited normal standing ability, with no obvious lesions observed in the tibial growth plate. Broilers in the TD group, induced by thiram, displayed marked motor dysfunction and difficulty standing. Observation of the tibial joint revealed white cartilage plugs (outlined by white dashed boxes), consistent with typical tibial dyschondroplasia lesions (Figure 3A). Thiram challenge increased the tibial lesion scores, whereas sodium butyrate supplementation significantly reduced the lesion scores compared with the TD group, although the scores remained higher than those of the control group. Not all 70 broilers assigned to the TD group developed TD lesions, and the severity varied among affected individuals (Figure 3B). Figure 3C shows the mean lesion scores. The TD group had a much higher score than the CON and TSB groups (*p* < 0.05). The TSB group had a lower score than TD, but still a higher score than CON (*p* < 0.05). Figure 3D shows morphological differences in the tibial growth plates among the three groups. No obvious histopathological abnormalities were detected in the CON group; the growth plates exhibited a clear compartmentalized structure comprising the proliferative zone (PZ), hypertrophic zone (HZ), and maturation zone (MZ), accompanied by normal vascularization. In contrast, the TD group exhibited reduced vascularization within the growth plate, irregular distribution of chondrocytes, and a reduced number of chondrocytes with sparse distribution in the hypertrophic zone; marked nuclear dissolution was also observed in this group. In the TSB group, however, vascularization in the hypertrophic zone was increased, chondrocytes were arranged in an orderly manner, and the morphology of cells in the hypertrophic zone was largely intact, resembling the findings in the control group. Only a small number of chondrocytes with mild nuclear dissolution were observed in the TSB group, whereas such changes were rarely detected in the CON group.

### 3.3. Tibial Morphological Parameters and Serum Bone Metabolism Markers

According to the results presented in Table 1, significant variations were observed in tibial morphological parameters among the groups. Tibial length and weight in both the TD and TSB groups were notably lower in comparison with the CON group (*p* < 0.05). Tibial width was markedly lower in the TD group than in the CON and TSB groups, and the TSB group also showed a lower value than CON (*p* < 0.05). Growth plate width was significantly greater in the TD group versus CON, while sodium butyrate supplementation significantly decreased it, though it remained above CON (*p* < 0.05). The tibial growth plate index increased in TD broilers versus the controls (*p* < 0.05). This index was decreased in the TSB group, yet it was still notably higher in relation to the control group (*p* < 0.05). For serum bone metabolism indicators, the concentrations of Ca and ALP were markedly reduced in TD relative to CON (*p* < 0.05), whereas no notable difference in serum phosphorus content was observed among all groups (*p* > 0.05). In the TSB group, serum Ca and ALP levels were comparable to TD (*p* > 0.05) but lower than CON (*p* < 0.05).

### 3.4. Tibial Mineralization Parameters

Figure 4 presents the tibial mineralization parameters of the three experimental groups. As shown by radiographic analysis (Figure 4A), broilers in the TD group exhibited pronounced mineralization defects, characterized by decreased bone density and distinct lesion areas highlighted in red. In contrast, such abnormalities were not detected in either the CON or TSB groups. Quantitative analyses (Figure 4B–D) indicated that bone mineral density was significantly lower in TD than in CON (*p* < 0.05), whereas TSB and CON showed no significant difference (*p* > 0.05). Conversely, both bone mineral content and tibial bone volume were lower in TD and TSB than CON (*p* < 0.05). With respect to bone mineral density, TSB had significantly higher values than TD (*p* < 0.05); however, bone mineral content and tibial bone volume did not differ significantly between these two groups (*p* > 0.05). Analysis of bone mineral composition showed no significant intergroup differences in tibial ash content (Figure 4E). Tibial calcium levels were lower in TD than CON (*p* < 0.05), while TSB was intermediate and comparable to both (Figure 4F). Tibial phosphorus tended to be higher in CON than in TD and TSB, although the differences did not reach statistical significance (Figure 4G).

### 3.5. Oxidative Stress and Antioxidant Status in the Ileal Mucosa and Serum

The antioxidant status of the ileal mucosa and serum is presented in Table 2. In the ileal mucosa, TD induction was associated with increased oxidative stress, as reflected by elevated MDA and reduced SOD and GSH-Px activities compared with the CON group (*p* < 0.05). Sodium butyrate supplementation significantly reduced the ileal MDA content to levels comparable to CON and significantly lower than TD (*p* < 0.05). Ileal GSH-Px in the TSB group was intermediate and did not differ significantly from either CON or TD (*p* > 0.05). Ileal mucosal T-AOC was comparable across groups (*p* > 0.05). In serum, TD-induced broilers exhibited the most severe oxidative imbalance, characterized by the highest ROS and MDA levels and the lowest activities of SOD, GSH-Px, and T-AOC (*p* < 0.05). Sodium butyrate supplementation partially alleviated systemic oxidative stress, as evidenced by reduced serum MDA concentrations and enhanced GSH-Px activity. In the TSB group, SOD and T-AOC levels were similar to those in CON and TD, whereas serum ROS levels in the TSB group were significantly lower than those in the TD group but remained higher than in the CON group (*p* < 0.05).

### 3.6. Effects of Sodium Butyrate on Mitochondrial Function-Related Parameters in the Ileal Mucosa

Thiram challenge markedly impaired mitochondrial function in the ileal mucosa of broilers (Figure 5). Mitochondrial membrane potential (MMP; Figure 5A) was reduced in TD relative to CON (*p* < 0.05), whereas sodium butyrate supplementation restored MMP to levels comparable to CON. Ileal ROS levels (Figure 5B) and superoxide anion (O_2_^•−^; Figure 5C) were higher in TD than CON (*p* < 0.05). Dietary sodium butyrate reduced both ROS and O_2_^•−^ levels compared with TD (*p* < 0.05), with no difference from CON. Meanwhile, thiram challenge reduced the levels of mitochondrial respiratory chain complexes I (MRCC I; Figure 5D) and III (MRCC III; Figure 5E) in the ileal mucosa (*p* < 0.05). Sodium butyrate supplementation increased the MRCC I levels compared with TD (*p* < 0.05), resulting in values comparable to CON, whereas MRCC III in the TSB group was intermediate and did not differ from either CON or TD.

### 3.7. Linear Regression Analyses Between Oxidative Stress Markers and Tibial Development Parameters in Broilers

To further clarify the relationship between oxidative stress and tibial development, linear regression analyses were performed between the serum and ileal oxidative stress markers and tibial parameters (Figure 6A–H). All tested oxidative stress markers (serum and ileal ROS and MDA) showed significant positive correlations with the growth plate index and significant negative correlations with BMD (*p* < 0.05).

### 3.8. Short-Chain Fatty Acid Concentrations in the Ileum

Table 3 summarizes the concentrations of short-chain fatty acids among the experimental groups. Acetic acid concentration was reduced in TD relative to CON (*p* < 0.05), while TSB did not differ from CON or TD (*p* > 0.05). Among the measured short-chain fatty acids, butyric acid was present at significantly greater levels in the TSB group than in both the CON and TD groups (*p* < 0.05). In contrast, the three groups did not differ significantly in propionic, isobutyric, or valeric acid concentrations (*p* > 0.05).

### 3.9. Ileal Microbiota Composition

As illustrated in Figure 7, dietary sodium butyrate altered the ileal microbial diversity and global community composition in broilers. Analysis of α-diversity (Figure 7A) showed that there were no significant differences among the CON, TD, and TSB groups in the ACE, Chao1, Shannon, or Simpson indices (*p* > 0.05). ASV-level PCoA revealed a clear separation among the three treatments (*R* = 0.2410, *p* = 0.039; Figure 7B). The initial two principal coordinates explained 26.4% (PC1) and 15.8% (PC2) of the total variance, respectively, suggesting that the dietary treatments substantially influenced the composition of the ileal microbiota. At the phylum level (Figure 7C,D), Bacillota dominated the microbial communities in all groups; however, its relative abundance was significantly lower in TD and TSB broilers versus CON (*p* < 0.05). In contrast, Pseudomonadota abundance differed significantly among all three groups, with CON having the lowest abundance and TD the highest (*p* < 0.05). Additionally, Actinomycetota was more abundant in TSB than TD (*p* < 0.05) and comparable to CON. Genus-level analysis demonstrated pronounced shifts in ileal microbial composition among dietary treatments (Figure 7E,F). *Ligilactobacillus* abundance declined in broilers receiving the TD diet compared with the controls, whereas sodium butyrate supplementation increased its relative abundance compared with the TD group, restoring levels comparable to those observed in the CON group (*p* < 0.05). *Lactobacillus* abundance was markedly elevated in the TSB relative to CON and TD (*p* < 0.05). Conversely, *Limosilactobacillus* showed its lowest abundance in the TD group, with higher levels detected in both the CON and TSB groups (*p* < 0.05). TD treatment also resulted in a marked enrichment of *Escherichia–Shigella* relative to the controls (*p* < 0.05); although this genus was partially reduced by sodium butyrate supplementation, its abundance in the TSB group remained higher than that in the CON group but significantly lower than that in the TD group (*p* < 0.05). In addition, *Blautia* and *Mediterraneibacter* were significantly more abundant in the TSB group than in either the CON or TD groups (*p* < 0.05).

### 3.10. Gene Expression in the Ileal Mucosa and Tibial Growth Plate

In the ileal mucosa, the TD group exhibited distinctly lower mRNA expression of *Nrf2* and *HO-1* in comparison with the CON group (*p* < 0.05) (Figure 8A,B). With dietary sodium butyrate supplementation, the *Nrf2* mRNA levels in the TSB group increased versus TD (*p* < 0.05) and were comparable to CON (*p* > 0.05). *HO-1* expression was significantly lower in TD than in CON (*p* < 0.05), whereas TSB exhibited intermediate levels that did not differ from either group (*p* > 0.05). *Nrf2* mRNA was markedly lower in TD than in CON and TSB in the tibial growth plate (*p* < 0.05), and CON and TSB had similar levels (*p* > 0.05) (Figure 8C). *HO-1* mRNA was downregulated in TD vs. CON, while TSB showed intermediate expression, differing significantly from both (*p* < 0.05) (Figure 8D). Consistent with the alterations in antioxidant-related genes, bone metabolism-related gene expression was disrupted in the TD group, as indicated by a significant decrease in *OPG* mRNA expression and a concomitant increase in *RANKL* mRNA expression (*p* < 0.05) (Figure 8E,F), leading to a markedly reduced OPG/RANKL mRNA ratio (*p* < 0.05) (Figure 8G). Relative to the TD group, sodium butyrate supplementation in the TSB group elevated *OPG* and reduced *RANKL* expression (*p* < 0.05). Consequently, the OPG/RANKL mRNA ratio was significantly higher than in TD (*p* < 0.05), yet still lower than the corresponding value in the CON group. Among genes associated with chondrogenesis and osteogenesis, the mRNA level of *COL2A1* was significantly lower in TD than CON (*p* < 0.05) (Figure 8H). Sodium butyrate supplementation significantly increased *COL2A1* expression relative to the TD group (*p* < 0.05). Furthermore, the *RUNX2* mRNA levels were similar across all groups (Figure 8I).

### 3.11. Correlation Analysis Among Ileal Microbiota, Oxidative Stress, and Tibial Development

The results of the Mantel test indicate that there is a significant overall correlation between dominant bacterial genera in the ileum and indicators of oxidative stress, antioxidant status, and tibial development (Figure 9). In particular, *Ligilactobacillus*, *Lactobacillus*, and *Mediterraneibacter* were significantly correlated with serum and ileal ROS and MDA levels (*p* < 0.05), and exhibited moderate to strong correlations (r ≥ 0.2) with ileal antioxidant enzyme activities (SOD) and indicators of the Nrf2/HO-1 signaling pathway. Furthermore, dominant ileal bacterial genera were significantly correlated with the OPG/RANKL ratio, *COL2A1* gene expression in the tibia, tibial bone mineral density (BMD) and growth plate index. These results suggest that the microbiota may participate in the development of tibial dyschondroplasia by regulating oxidative stress status and pathways related to bone metabolism. Pearson correlation analysis revealed that serum and ileal oxidative stress markers (ROS, MDA) were significantly positively correlated with the tibial growth plate index while being significantly negatively correlated with tibial BMD and the OPG/RANKL ratio (*p* < 0.05). Furthermore, *Nrf2* and *HO-1* expression in the ileum and tibia correlated positively with SOD and GSH-Px activities, and negatively with ROS and MDA levels (*p* < 0.05). A positive correlation was found between the OPG/RANKL ratio and tibial BMD and *COL2A1* expression, suggesting a possible link between oxidative stress and an imbalance in the OPG/RANKL pathway. In the Pearson correlation analysis, ileal butyrate levels did not show significant linear correlations with several oxidative stress and antioxidant-related indicators (*p* > 0.05). However, in the Mantel test, the butyrate-producing bacterium *Mediterraneibacter* showed a significant overall correlation with the ileal butyrate levels and markers of the Nrf2/HO-1 signaling pathway, suggesting that butyrate may participate in regulating oxidative stress and bone metabolism through indirect, gut microbiota-mediated mechanisms.

## 4. Discussion

The present study provides evidence that dietary sodium butyrate alleviates thiram-induced TD in broilers. Our key findings indicate that sodium butyrate reshaped gut microbiota composition, enhanced intestinal and systemic antioxidant capacity, preserved mitochondrial function in the ileal mucosa, and improved bone metabolism as reflected by restored OPG/RANKL ratio and enhanced *COL2A1* expression. These results suggest that sodium butyrate exerts protective effects through a coordinated gut–bone axis mechanism involving redox regulation and mitochondrial preservation.

### 4.1. Sodium Butyrate Improves Growth Performance and Skeletal Development in Broilers with TD

Thiram is a well-established inducer of TD that disrupts proximal tibial growth plate development by impairing chondrocyte differentiation and vascular invasion [41,42,43]. In the present study, broilers in the TD group showed reduced body weight, ADFI, and ADG. These changes were accompanied by typical TD manifestations, including difficulty standing and white cartilage lesions in the proximal tibia. These observations are consistent with previous reports on thiram-induced TD in broilers [44,45], verifying the successful induction of the TD model.

Butyrate serves as the primary energy substrate for intestinal epithelial cells and has been shown to enhance nutrient absorption and barrier function [46,47]. In our study, sodium butyrate supplementation (500 mg/kg) significantly improved BW, ADFI, and ADG in thiram-challenged broilers, particularly at day 21. This growth recovery coincided with reduced serum and ileal ROS/MDA and elevated SOD/GSH-Px activities, suggesting that the growth-promoting effect is not merely nutritional but also linked to the alleviation of oxidative stress, which is known to impair appetite and nutrient utilization in poultry [26,27]. Histological and morphological analyses further showed that sodium butyrate alleviated growth plate lesions, improved chondrocyte organization, and promoted vascular distribution. These effects may be related to improved redox homeostasis, which is important for chondrocyte differentiation and vascular invasion within the growth plate [48]. This aligns with earlier reports demonstrating that SCFAs improve growth plate function by promoting cartilage vascularization [31]. In addition, sodium butyrate supplementation improved tibial morphological parameters and restored bone mineral density (BMD) to a level comparable to that of the CON group. However, BMC and tibial bone volume in the TSB group did not fully recover to the control levels, suggesting partial rather than complete restoration of skeletal structure. Similar incomplete recovery of certain bone parameters following nutritional intervention has also been documented in earlier TD studies [49], which may be related to the relatively longer time required for bone mineralization and remodeling compared with the duration of the present intervention. Further studies with longer intervention periods or dose-gradient designs are needed to validate this possibility.

### 4.2. Gut Microbiota Remodeling Is Associated with Improvements in Redox Homeostasis

The gut microbiota modulates host redox regulation and skeletal health [50,51]. Microbial dysbiosis may impair intestinal barrier function, increase inflammatory and oxidative burden, and indirectly disturb bone homeostasis [52,53,54]. In the present study, thiram-induced TD was associated with alterations in ileal microbial community composition, including reduced Bacillota abundance and enrichment of Pseudomonadota and *Escherichia–Shigella*, indicating ileal dysbiosis. This compositional shift, characterized by a decline in Bacillota and overgrowth of Pseudomonadota, is a hallmark of gut barrier impairment and endotoxin leakage [55].

Sodium butyrate supplementation reshaped the ileal microbiota by increasing several potentially beneficial genera, including *Ligilactobacillus*, *Lactobacillus*, *Blautia*, and *Mediterraneibacter*. Meanwhile, ileal butyrate concentration was increased, whereas acetate showed a partial numerical recovery. These findings corroborate earlier reports that sodium butyrate can modulate gut microbiota and stimulate SCFA production [28,56]. Among the enriched taxa, *Mediterraneibacter* is recognized as a butyrate-producing genus [57], suggesting that sodium butyrate may not only act as an exogenous dietary metabolite, but also support an intestinal environment favorable for endogenous butyrate-related metabolism.

Importantly, correlation analyses indicated significant associations between microbial alterations and oxidative stress markers, antioxidant indices, Nrf2/HO-1 mRNA levels, and skeletal parameters. Although ileal butyrate concentration was not significantly correlated with several individual antioxidant indices, the Mantel test supported an overall association between butyrate-related bacteria and the antioxidant response. These findings suggest that gut microbiota remodeling may be closely associated with improvements in redox status and may play a role in the protective effects of sodium butyrate against TD.

### 4.3. Sodium Butyrate Alleviates Oxidative Stress Accompanied by Upregulation of Nrf2 and HO-1 Expression

Given the observed associations between microbiota remodeling and antioxidant-related indices, oxidative stress appears to be a key downstream process in sodium butyrate-mediated protection against TD. Oxidative stress is a central pathological driver of TD onset and progression [58]. Mitochondria are the primary endogenous source of intracellular ROS; impaired mitochondrial electron transport chain integrity triggers excess superoxide anion (O_2_^•−^) accumulation and depolarized mitochondrial membrane potential (MMP), initiating intracellular oxidative cascade reactions [59,60]. In the present thiram-induced TD model, ileal mucosal mitochondria exhibited severe functional damage, manifested as reduced MMP, suppressed respiratory chain complex I/III activity, and elevated O_2_^•−^ and tissue ROS concentrations. The Nrf2/HO-1 pathway acts as the core downstream antioxidant defense axis to eliminate excess ROS and restore cellular redox homeostasis [12]. Previous studies have shown that oxidative insults can suppress Nrf2 activation and downstream antioxidant gene expression, thereby aggravating tissue injury [61]. Linear regression analysis further quantified that these intestinal mitochondrial-derived oxidative mediators were positively correlated with tibial growth plate hypertrophy and negatively correlated with bone mineral density, confirming intestinal mitochondrial dysfunction as a potential upstream contributor of systemic oxidative imbalance and subsequent skeletal developmental defects.

In the present study, TD broilers exhibited increased ROS and MDA levels in serum and ileal mucosa, together with reduced SOD and GSH-Px activities. These biochemical changes were accompanied by decreased *Nrf2* and *HO-1* mRNA expression, supporting the presence of marked oxidative imbalance in thiram-induced TD. This is consistent with findings from ethanol-induced liver injury models in mice, in which oxidative stress suppresses Nrf2/HO-1 signaling and weakens antioxidant defenses [62]. Collectively, these results are consistent with a cascade model in which thiram-induced mitochondrial dysfunction drives ROS overproduction, which in turn impairs Nrf2/HO-1-mediated antioxidant defenses, leading to growth plate oxidative damage; sodium butyrate supplementation alleviated oxidative damage, as reflected by reduced ROS and MDA levels in both the serum and ileal mucosa, partial restoration of antioxidant enzyme activities, and increased Nrf2/HO-1 mRNA expression. Notably, sodium butyrate also preserved mitochondrial integrity, as evidenced by restored MMP, reduced O_2_^•−^ accumulation, and increased MRCC I activity. Our results indicate that sodium butyrate may alleviate oxidative stress through a dual mechanism: reducing mitochondrial ROS production at the source while simultaneously enhancing Nrf2/HO-1-mediated antioxidant clearance. This dual pattern is consistent with previous evidence that sodium butyrate can not only suppress mitochondrial ROS overproduction and preserve mitochondrial function under oxidative stress conditions, but also activate the Nrf2/HO-1-mediated antioxidant defense system [63,64]. Similarly, Malhi et al. demonstrated that SCFAs can enhance antioxidant capacity in broilers via Nrf2-dependent pathways [65]. Notably, sodium butyrate restored *Nrf2* mRNA expression to a level comparable to that of the CON group, whereas *HO-1* expression in the tibial growth plate showed only partial recovery. This discrepancy may reflect post-transcriptional regulation of Nrf2 protein stability or differential promoter sensitivity to butyrate-mediated histone deacetylase inhibition [66,67]. Similarly, MRCC III activity in the TSB group showed only a moderate and non-significant recovery, suggesting that not all mitochondrial complexes or downstream antioxidant effectors respond uniformly to butyrate within the current intervention timeframe.

### 4.4. Sodium Butyrate Partially Restores Skeletal Homeostasis in TD

Skeletal homeostasis depends on coordinated bone remodeling, mineralization, and normal chondrocyte maturation. The OPG/RANKL system is widely used as an indicator of osteoclast-related bone remodeling [68]. OPG, as a decoy receptor for RANKL, inhibits RANKL–RANK signaling, thereby limiting osteoclast differentiation and bone resorption [69,70]. Prior investigations have demonstrated that oxidative stress can promote *RANKL* expression and inhibit *OPG* via the NF-κB and MAPK pathways, thereby driving enhanced bone resorption [71,72].

In the present study, TD broilers showed reduced *OPG* expression, increased *RANKL* expression, and a lower OPG/RANKL ratio in the tibial growth plate, consistent with previous observations in TD broilers [73]. These molecular changes were paralleled by reduced serum Ca and ALP levels, further supporting disturbed skeletal metabolism. Sodium butyrate partially reversed these changes, suggesting an improvement in bone metabolic status. Protective effects of short-chain fatty acids on bone homeostasis have been reported in other animal models [74,75]. Consistently, fermented calcium butyrate has been shown to protect bone homeostasis in post-peak laying hens by reducing osteoclast differentiation and bone resorption [76]. Furthermore, studies using *Anaerostipes caccae* supplementation in a chicken model demonstrated that butyrate-producing bacteria can enhance bone development and maintain bone homeostasis through the gut–bone axis [77].

In conjunction with the oxidative stress results, these findings suggest that sodium butyrate may improve skeletal homeostasis, at least in part, through the restoration of redox balance, with changes in the OPG/RANKL ratio serving as supportive evidence rather than an independent mechanistic pathway [78]. In addition, *COL2A1*, a key marker of chondrogenesis, was downregulated in the TD group but increased after sodium butyrate supplementation. This is consistent with reports that icariin promotes chondrocyte differentiation and reduces the incidence of TD by regulating P2RX7 in a thiram-induced tibial dyschondroplasia model in broilers [35]. In contrast, *RUNX2* expression did not differ significantly among groups. The differential responsiveness of *COL2A1* (restored by butyrate) and *RUNX2* (unchanged) indicates that sodium butyrate primarily promotes chondrocyte matrix synthesis rather than driving terminal osteoblast differentiation within the growth plate—a notion supported by our histological findings of improved chondrocyte organization and vascular invasion, as well as the unchanged serum phosphorus levels across groups. Overall, the skeletal benefits of sodium butyrate appear to be associated with gut microbiota remodeling and the restoration of redox homeostasis, which may collectively contribute to improved growth plate structure and tibial development.

Several limitations should be acknowledged. First, our data primarily revealed associations rather than causal relationships; the proposed gut–bone axis mechanism remains a hypothesis that warrants further investigation. Second, the partial recovery of certain parameters suggests that the current supplementation protocol may not be optimal, and future studies with refined designs could help clarify the dose and duration dependence. Third, the findings were obtained under controlled experimental conditions; their translation to commercial production settings should be interpreted with caution.

## 5. Conclusions

In conclusion, dietary sodium butyrate alleviated thiram-induced tibial dyschondroplasia in broilers, in association with improvements in gut microbiota composition, redox homeostasis, and bone metabolism. These protective effects were linked to attenuated oxidative damage, enhanced Nrf2/HO-1-related gene expression, and partial restoration of bone remodeling. By improving redox balance and supporting cartilage integrity, sodium butyrate is a promising nutritional approach for TD prevention, supporting an important involvement of oxidative stress in TD pathogenesis and an interaction between intestinal homeostasis and skeletal alterations.

## Figures and Tables

**Figure 1:**
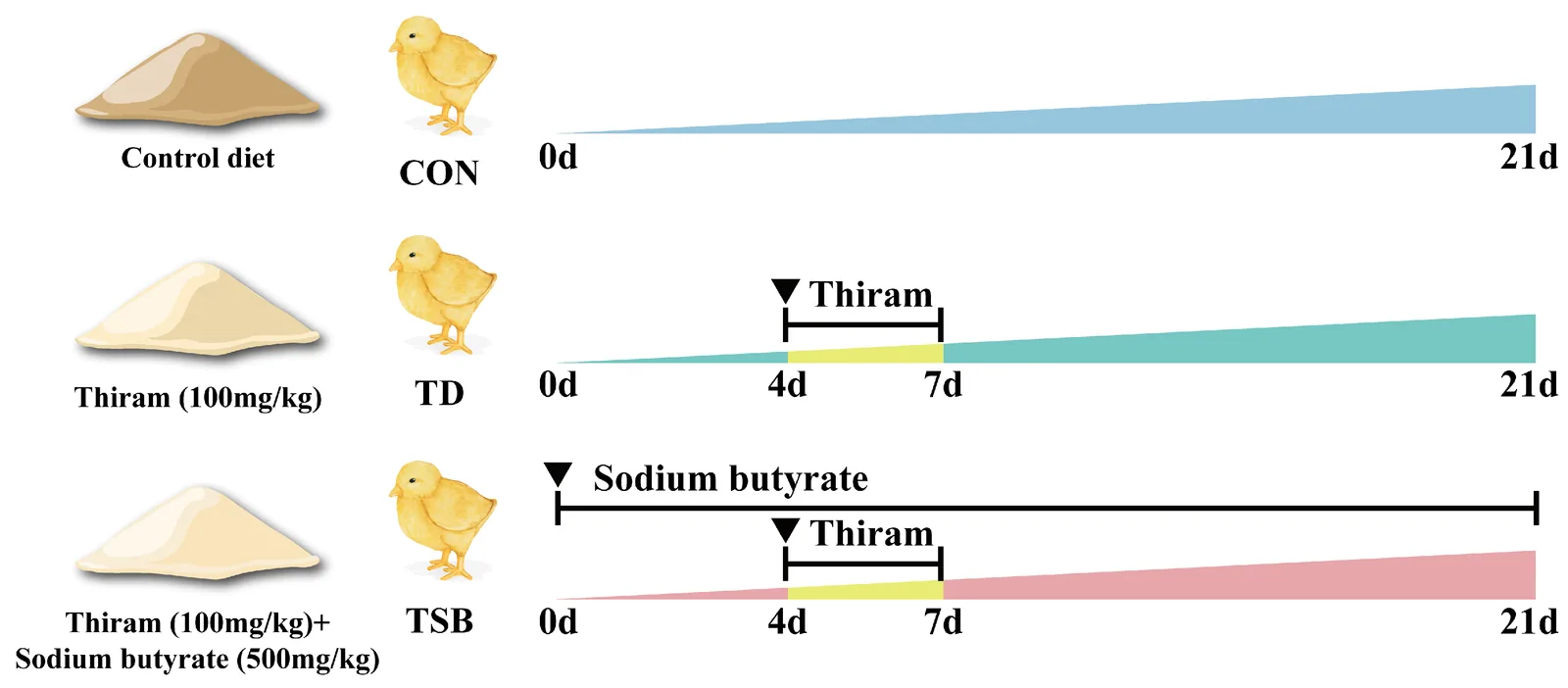
Experimental design schematic for the intervention against TD in broilers.

**Figure 2:**
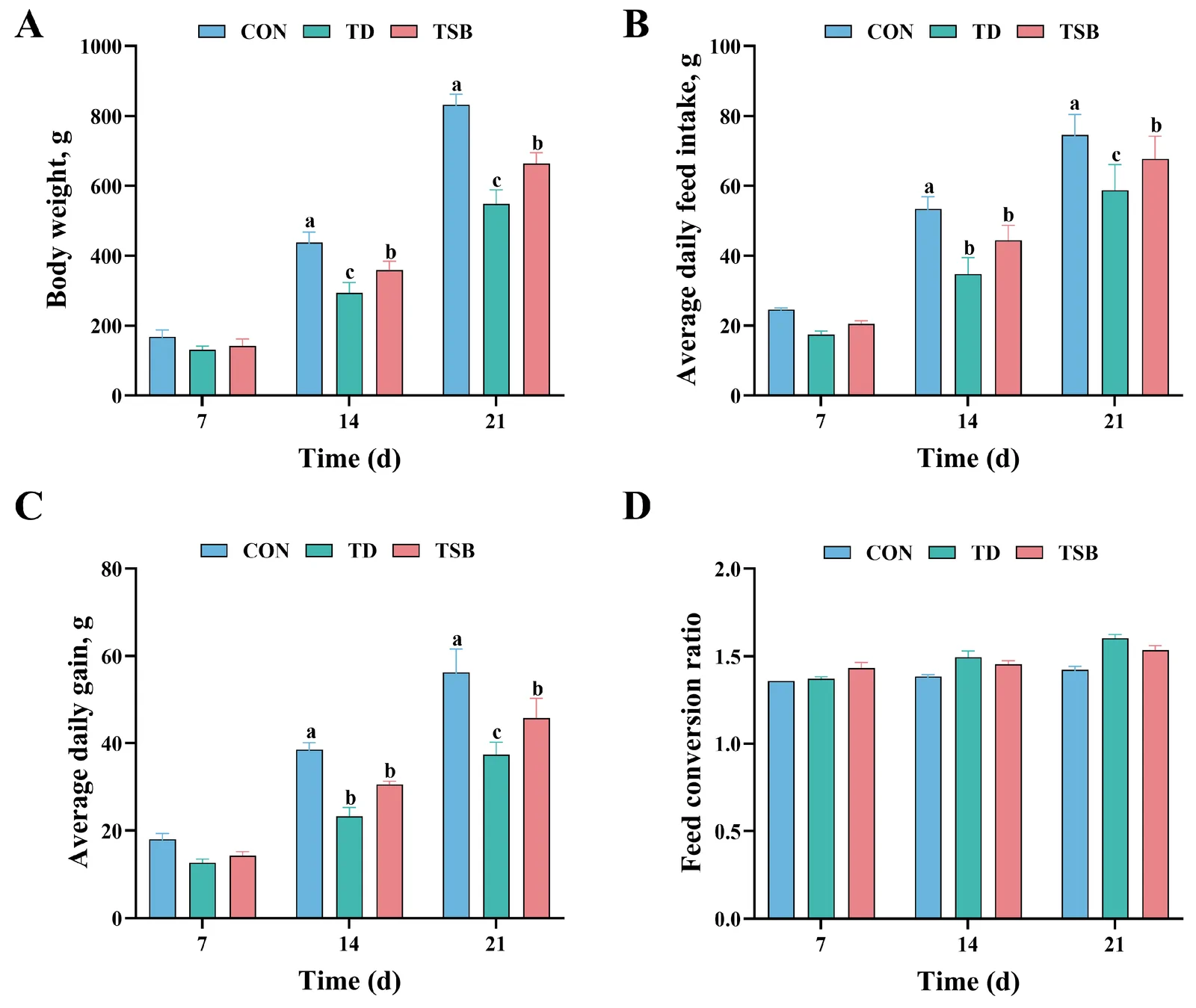
Effects of thiram challenge and dietary sodium butyrate supplementation on growth performance in broilers. (**A**) BW, (**B**) ADFI, (**C**) ADG, and (**D**) FCR. Body weight was recorded on days 7, 14, and 21; ADFI, ADG, and FCR were calculated for the intervals 0–7, 7–14, and 14–21. Values are mean ± SEM (*n* = 7 pens). Different letters indicate significant differences (*p* < 0.05).

**Figure 3:**
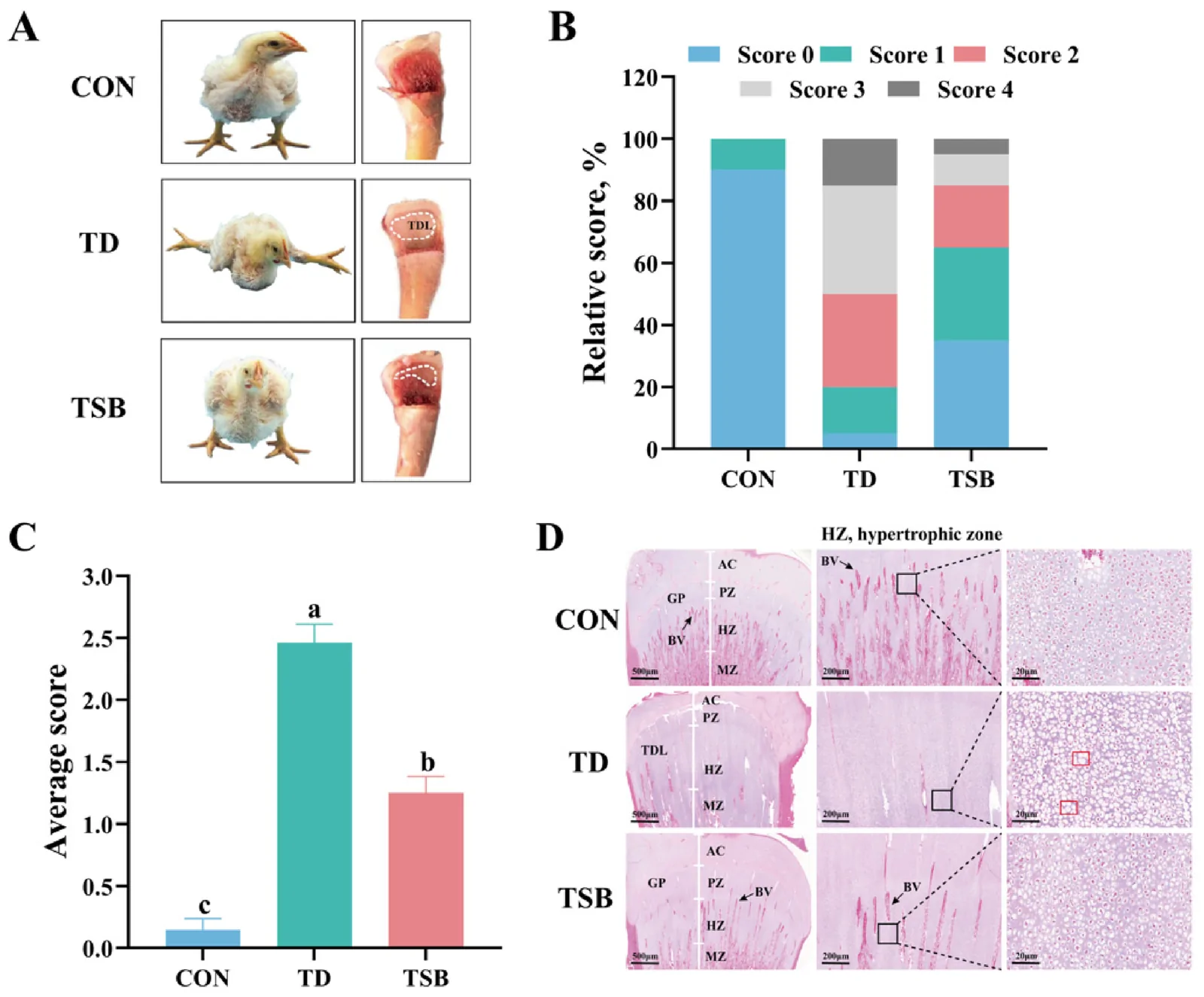
Clinical observations of thiram-induced tibial dyschondroplasia (TD). (**A**) Representative images of broilers and tibial joints in the CON, TD, and TSB groups. The tibial dyschondroplasia lesion (TDL) is outlined by a white dashed box in the TD group. (**B**) Relative distribution of lesion scores among groups. (**C**) Average clinical score of tibial lesions. Values are mean ± SEM (*n* = 7). Different letters denote *p* < 0.05. (**D**) Representative H&E-stained tibial growth plate sections from the CON, TD, and TSB groups. Low-magnification images show the overall structure of articular cartilage (AC) and the growth plate (GP), encompassing the PZ, HZ, and MZ. Medium- and high-magnification images focus on the hypertrophic zone to illustrate chondrocyte morphology and blood vessels (BV). Red boxes indicate karyolysis of chondrocytes in the TD group. Scale bars = 500 µm (low), 200 μm (medium), and 20 µm (high).

**Figure 4:**
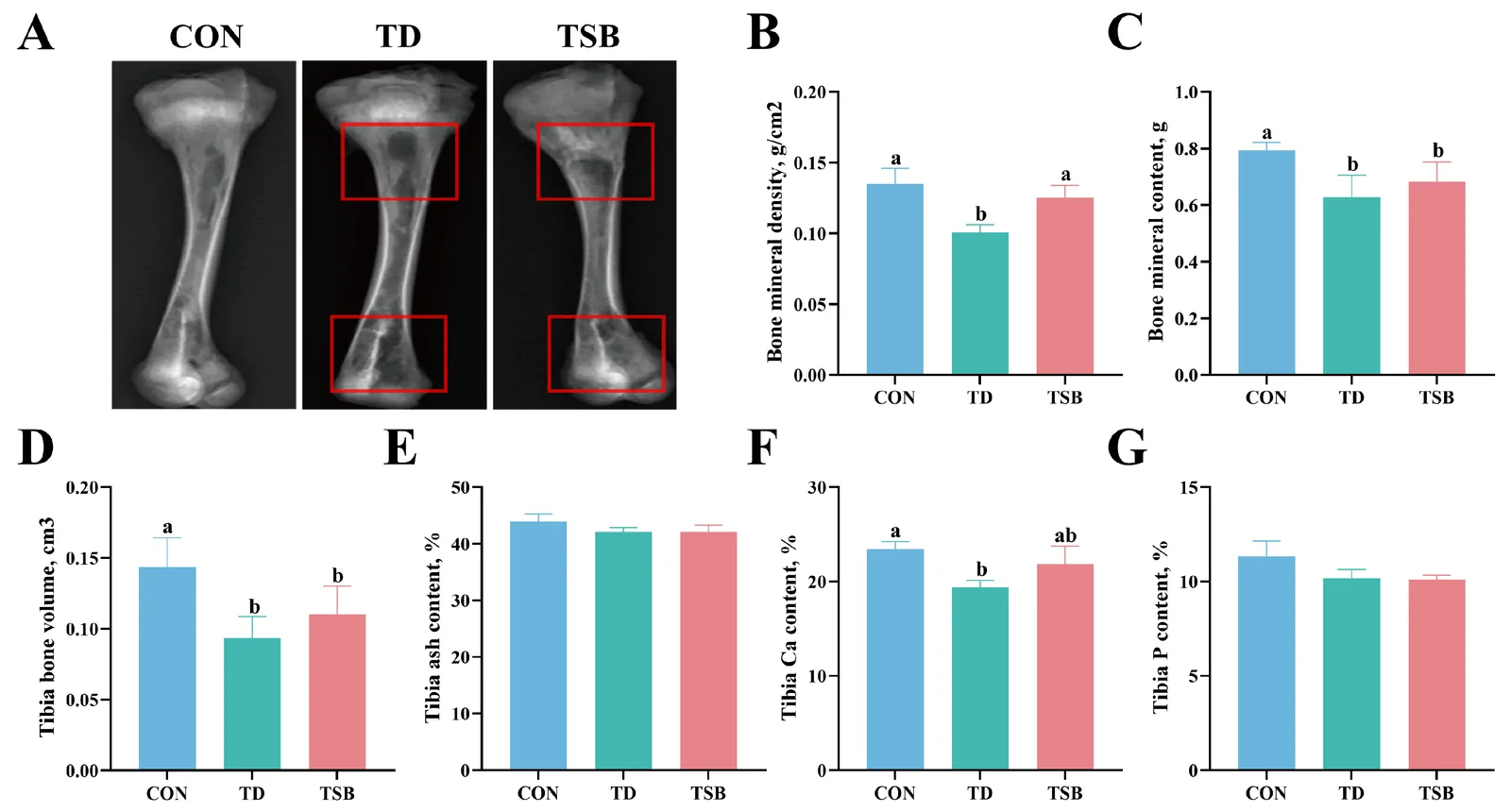
Effects of dietary sodium butyrate on the tibial mineralization parameters of broilers. (**A**) Representative X-ray images of tibiae from the CON, TD, and TSB groups. Red boxes indicate lesions in the TD group and alleviated regions in the TSB group. (**B**) BMD and (**C**) BMC. (**D**–**G**) Tibial bone volume, ash, Ca, and P contents. Values are mean ± SEM (*n* = 7). Different letters indicate *p* < 0.05; same letters indicate no significance.

**Figure 5:**
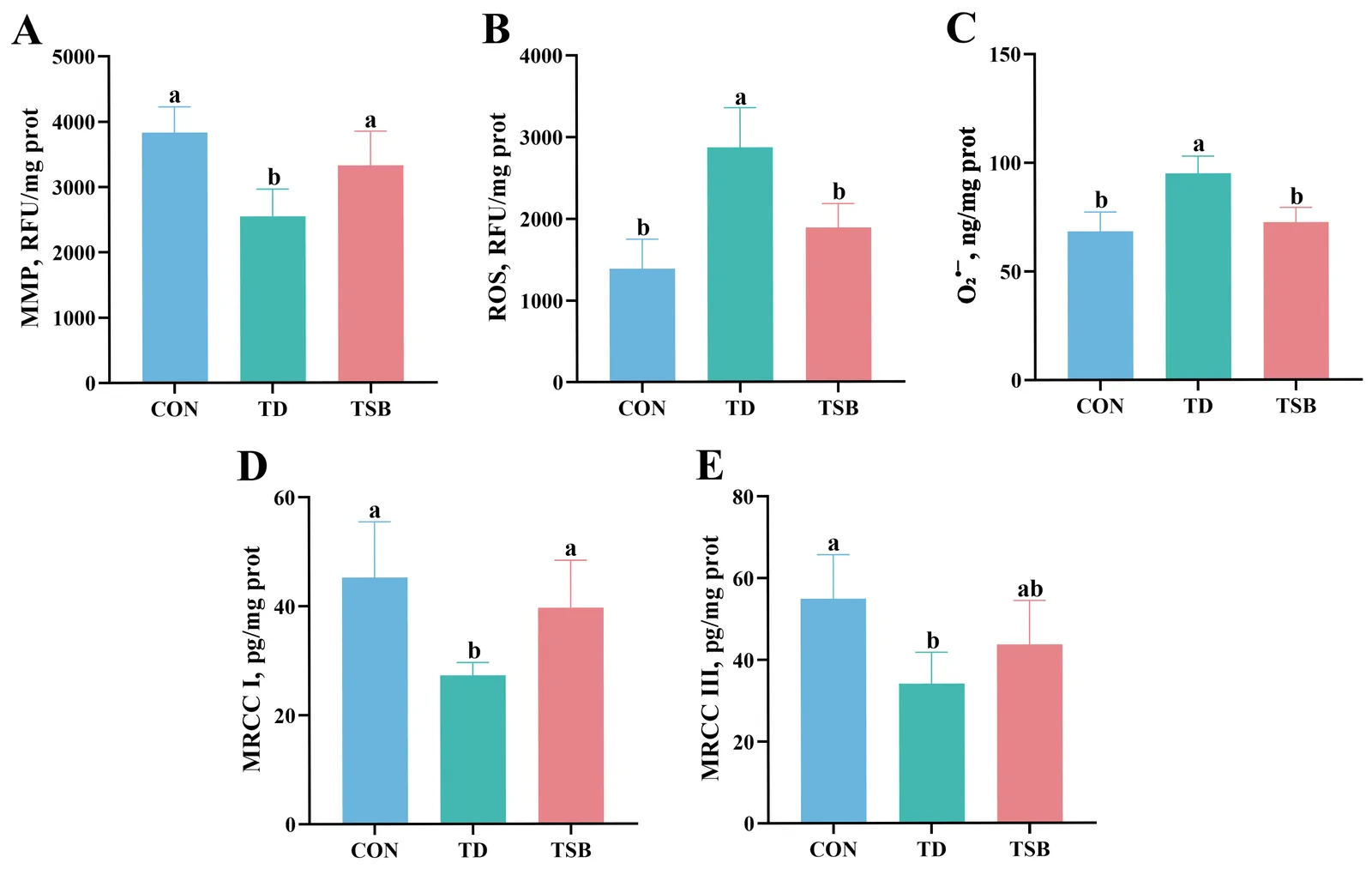
Effects of sodium butyrate on mitochondrial function-related parameters and oxidative stress indicators in the ileal mucosa of broilers challenged with thiram. (**A**) MMP, (**B**) ROS, (**C**) O_2_^•−^, (**D**) MRCC I, and (**E**) MRCC III. Values are mean ± SEM (*n* = 7). Different letters indicate *p* < 0.05; same letters indicate no significance.

**Figure 6:**
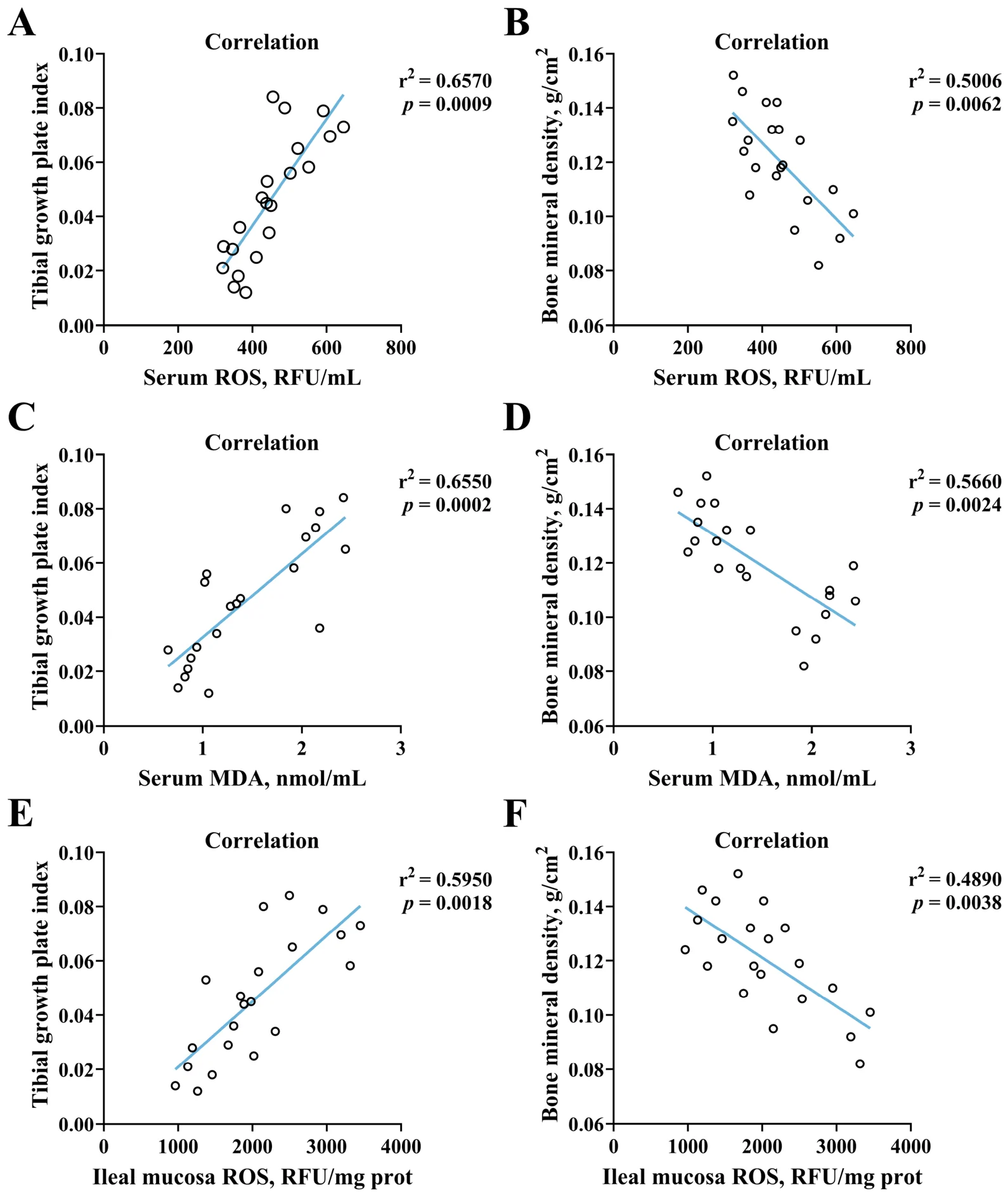

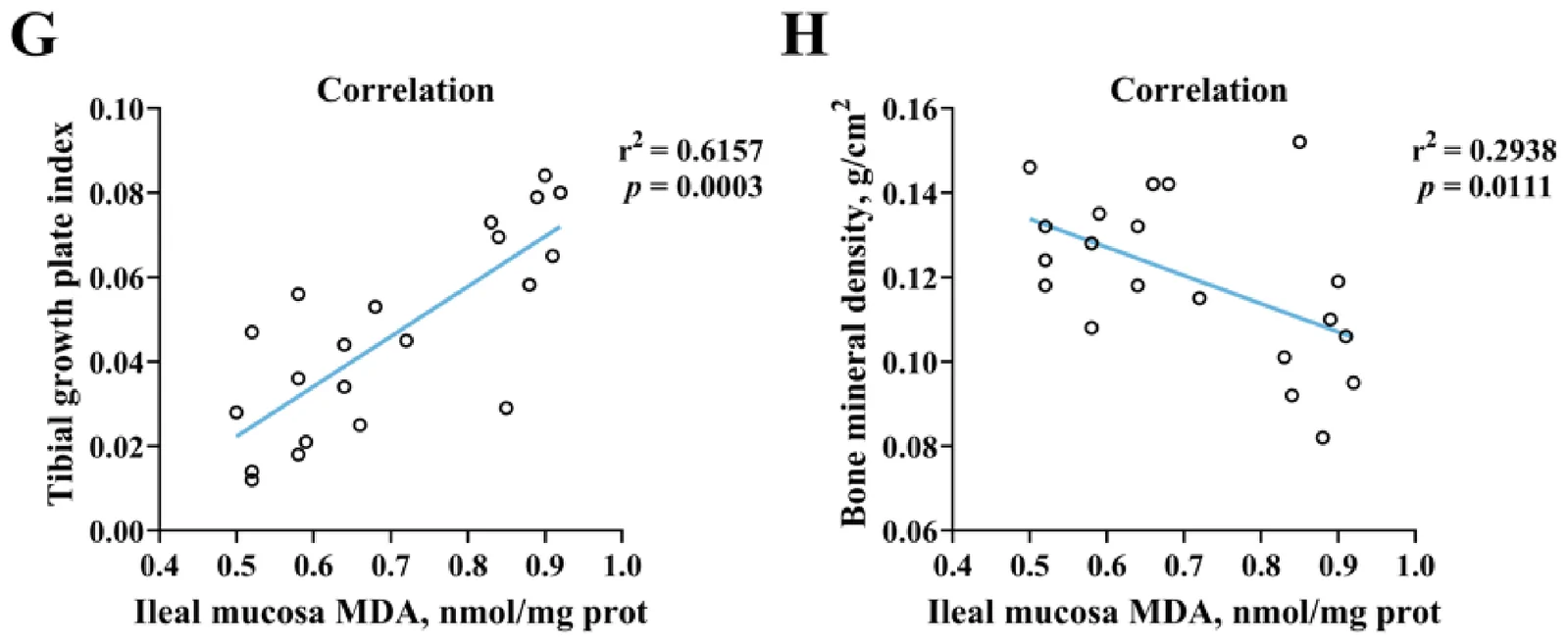
The correlations between oxidative stress markers and tibial development parameters were evaluated by linear regression analyses. (**A**–**H**) Linear regression analyses between the serum/ileal oxidative stress markers and tibial parameters (growth plate index and BMD). Each panel displays r^2^ and *p* values; significance was set at *p* < 0.05.

**Figure 7:**
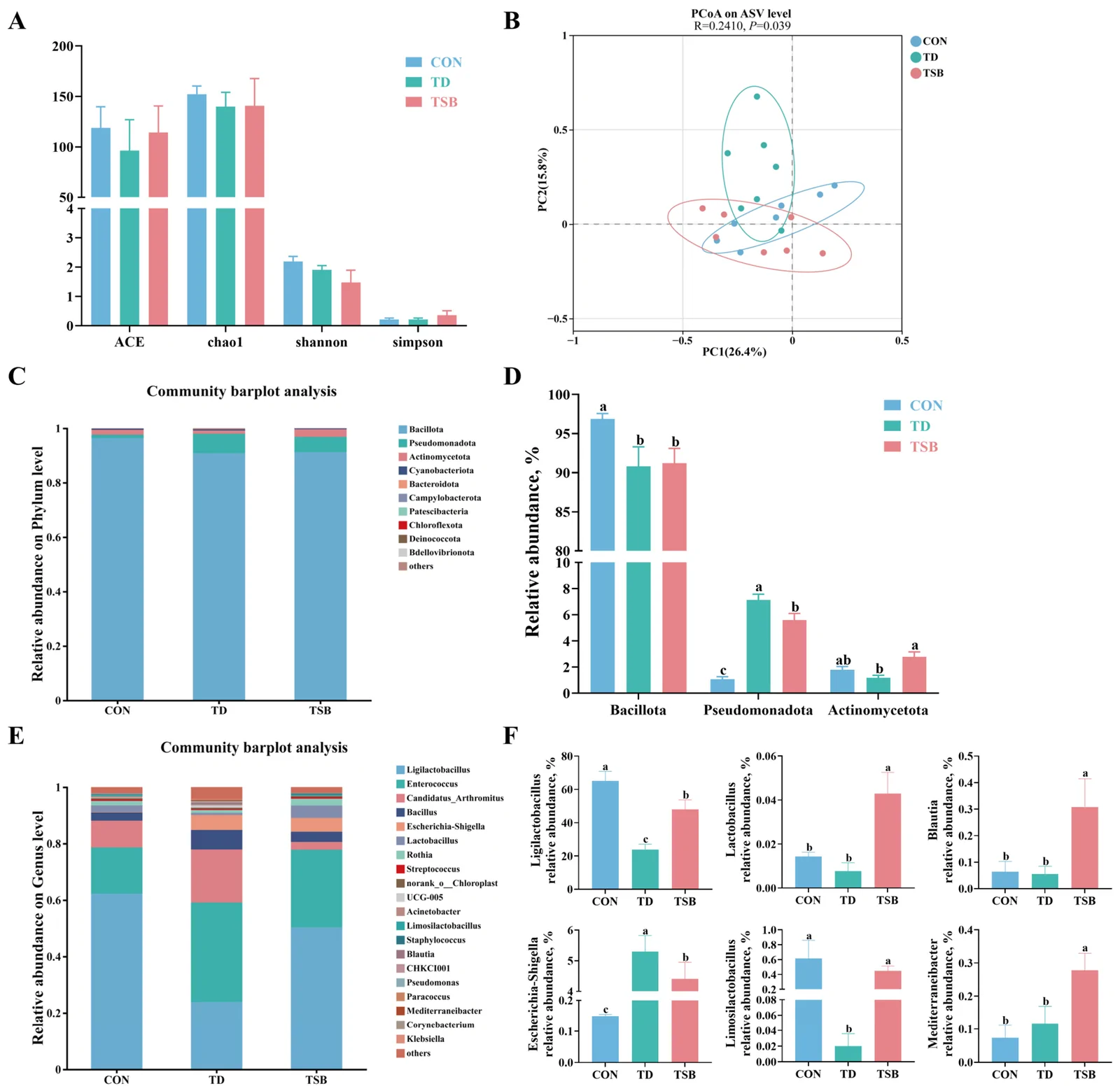
Effects of dietary treatments with sodium butyrate supplementation on ileal microbial diversity and community composition in broilers. (**A**) Alpha diversity indices including ACE, Chao1, Shannon, and Simpson. (**B**) Principal coordinate analysis (PCoA) performed using Bray–Curtis dissimilarity matrices at the ASV level. (**C**,**D**) Phylum-level bacterial composition and abundance. (**E**,**F**) Genus-level bacterial composition and abundance. Values are mean ± SEM (*n* = 7). Data are presented as CON (blue circles), TD (green circles), and TSB (red circles). Different letters indicate *p* < 0.05; same letters indicate no significance.

**Figure 8:**
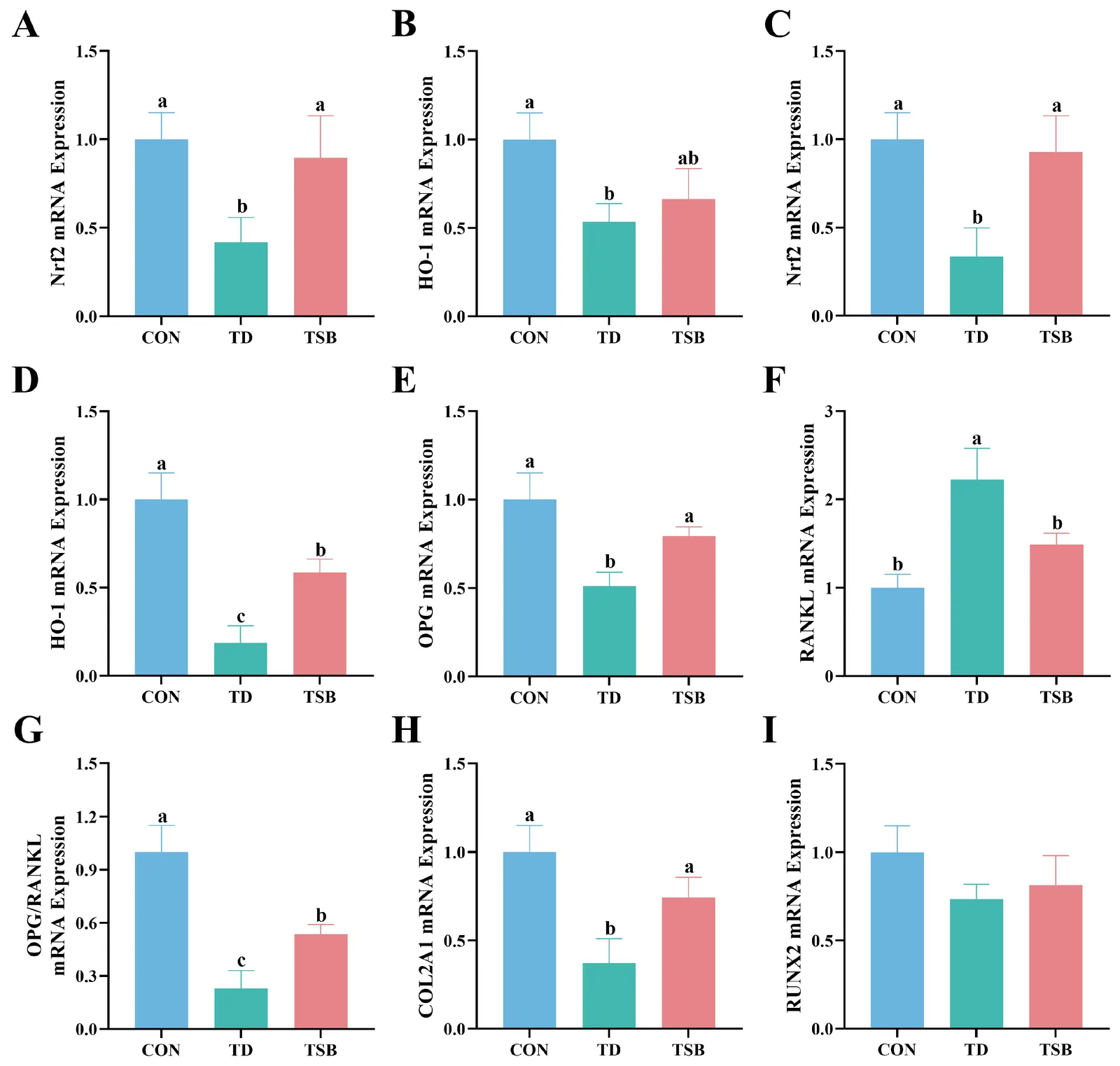
Effects of thiram and sodium butyrate supplementation on mRNA expression of oxidative stress-related genes in the ileal mucosa and bone metabolism-related genes in the tibial growth plate. (**A**,**B**) Ileal mucosal mRNA levels of *Nrf2* and *HO-1*; (**C**–**I**) tibial growth plate mRNA levels of *Nrf2*, *HO-1*, *OPG*, *RANKL*, OPG/RANKL mRNA ratio, *COL2A1*, and *RUNX2*. Values are mean ± SEM (*n* = 7). Different letters indicate *p* < 0.05; same letters indicate no significance.

**Figure 9:**
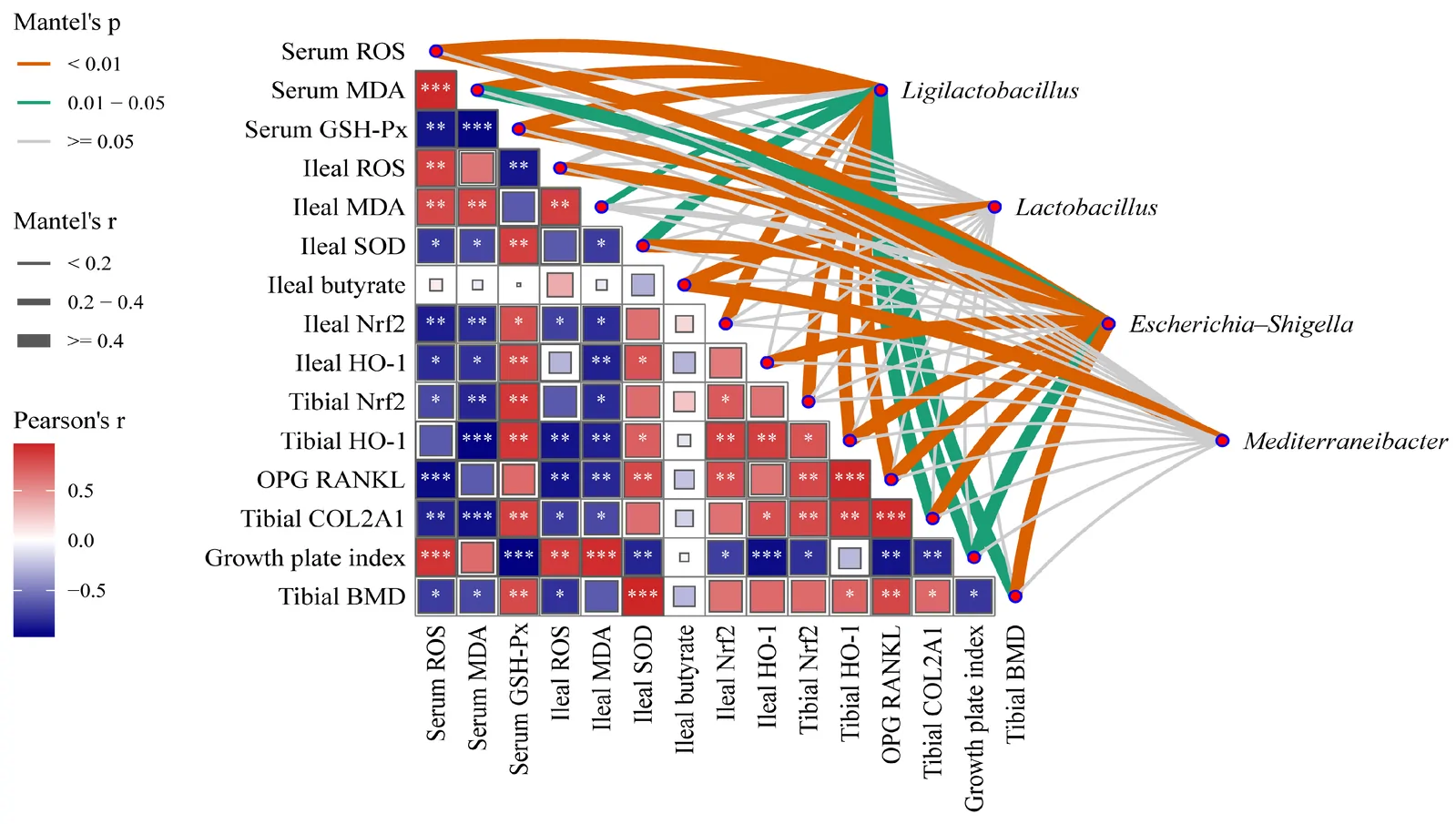
Correlation analysis between the ileal microbiota, oxidative stress parameters, and tibial development indices. Pearson correlation heatmap showing relationships among oxidative stress markers, antioxidant enzyme activities, Nrf2/HO-1-related transcripts, and tibial development parameters. Color gradient indicates Pearson’s r, with negative correlations represented by blue and positive correlations by red, while the degrees of statistical significance are marked as * *p* < 0.05, ** *p* < 0.01, and *** *p* < 0.001. Mantel test analysis illustrates the overall associations between dominant ileal bacterial genera (*Ligilactobacillus*, *Lactobacillus*, *Escherichia–Shigella*, and *Mediterraneibacter*) and physiological parameters. Line colors indicate significance levels (orange, *p* < 0.01; green, 0.01–0.05; grey, ≥0.05), and line thickness represents correlation strength (thin, r < 0.2; medium, 0.2–0.4; thick, ≥0.4). Each square represents the correlation coefficient between two variables, with the color gradient reflecting the value of Pearson’s r. Data were analyzed using Pearson correlation and the Mantel test (*n* = 7 per group).

**Table 1:** Tibial morphological indices and serum bone metabolism markers in broilers.

Items	Treatment			SEM	*p*-Value
	CON	TD	TSB		
**Tibial morphological parameters**					
Tibial length (mm)	69.54 ^a^	59.10 ^b^	61.26 ^b^	2.15	0.005
Tibial weight (g)	5.52 ^a^	4.45 ^b^	4.82 ^b^	0.12	<0.001
Tibial width (mm)	7.62 ^a^	6.04 ^c^	6.81 ^b^	0.25	0.025
Width of growth plate (mm)	1.37 ^c^	4.22 ^a^	2.77 ^b^	0.31	<0.001
Tibial growth plate index	0.020 ^c^	0.070 ^a^	0.040 ^b^	0.007	0.004
**Serum bone metabolism markers**					
Ca content (mmol/L)	2.13 ^a^	1.67 ^b^	1.84 ^b^	0.08	0.004
P content (mmol/L)	2.25	2.38	2.36	0.12	0.645
ALP (U/L)	1129.15 ^a^	985.62 ^b^	1011.38 ^b^	19.16	<0.001

Values are means with pooled SEM (*n* = 7). Different superscripts denote *p* < 0.05. Tibial length, tibial weight, tibial width, serum Ca, serum P, and ALP were analyzed by one-way ANOVA with Tukey’s post hoc test; growth plate width and tibial growth plate index were analyzed by the Kruskal–Wallis test with Dunn’s multiple comparisons. Abbreviations: SEM, standard error of the mean; CON, control; TD, thiram-induced TD; TSB, sodium butyrate-supplemented.

**Table 2:** Oxidative stress and antioxidant parameters in the ileal mucosa and serum.

Items	Treatment			SEM	*p*-Value
	CON	TD	TSB		
**Ileal mucosa oxidative stress and antioxidant indices**					
MDA (nmol/mg protein)	0.59 ^b^	0.85 ^a^	0.68 ^b^	0.05	0.011
SOD (U/mg protein)	28.91 ^a^	22.62 ^b^	24.29 ^b^	1.21	0.004
GSH-Px (U/mg protein)	7.11 ^a^	5.17 ^b^	5.64 ^ab^	0.61	0.045
T-AOC (U/mg protein)	0.38	0.21	0.25	0.08	0.190
**Serum oxidative stress and antioxidant indices**					
ROS (RFU/mL)	332.40 ^c^	551.57 ^a^	438.12 ^b^	34.91	<0.001
MDA (nmol/mL)	0.85 ^b^	2.14 ^a^	1.24 ^b^	0.17	0.001
SOD (U/mL)	555.12 ^a^	442.94 ^b^	485.62 ^ab^	27.17	0.017
GSH-Px (U/mL)	913.31 ^a^	785.57 ^c^	860.15 ^b^	10.29	<0.001
T-AOC (U/mL)	0.75 ^a^	0.48 ^b^	0.57 ^ab^	0.07	0.044

Values are means with pooled SEM (*n* = 7). Different letters indicate *p* < 0.05; same letters indicate no significance. Ileal SOD, ileal GSH-Px, serum SOD, serum GSH-Px, and serum T-AOC were analyzed by one-way ANOVA with Tukey’s post hoc test; ileal MDA, serum ROS, and serum MDA were analyzed by the Kruskal–Wallis test with Dunn’s multiple comparisons. Abbreviations: SEM, standard error of the mean; CON, control; TD, thiram-induced TD; TSB, sodium butyrate-supplemented; MDA, malondialdehyde; SOD, superoxide dismutase; GSH-Px, glutathione peroxidase; T-AOC, total antioxidant capacity; ROS, reactive oxygen species.

**Table 3:** Ileal short-chain fatty acid concentrations.

Items	Treatment			SEM	*p*-Value
	CON	TD	TSB		
Acetic acid (μmol/g)	41.71 ^a^	38.46 ^b^	39.60 ^ab^	1.56	0.048
Propionic acid (μmol/g)	2.25	2.03	2.31	0.45	0.626
Butyric acid (μmol/g)	5.89 ^b^	6.22 ^b^	8.57 ^a^	0.86	<0.001
Isobutyric acid (μmol/g)	0.32	0.22	0.18	0.05	0.124
Valeric acid (μmol/g)	0.85	0.94	1.05	0.10	0.211

Values are means with pooled SEM (*n* = 7). Different letters indicate *p* < 0.05; same letters indicate no significance. All variables (acetic acid, propionic acid, butyric acid, isobutyric acid, and valeric acid) were analyzed by the Kruskal–Wallis test with Dunn’s multiple comparisons. Abbreviations: SEM, standard error of the mean; CON, control; TD, thiram-induced TD; TSB, sodium butyrate-supplemented.

## Data Availability

The 16S rRNA sequencing data have been deposited in the NCBI SRA under BioProject PRJNA1458744. Other data are available within the article and Supplementary Materials.

## Supplementary Materials

The following supporting information can be downloaded at: https://www.mdpi.com/article/10.3390/antiox15101302/s1, Table S1: Ingredient and nutrient composition of the basal diet used in this study; Table S2: Primers used for the RT-qPCR.

## Abbreviations

AC, Articular cartilage; ACE, Abundance-Based Coverage Estimator; ADFI, Average daily feed intake; ADG, Average daily gain; ALP, Alkaline phosphatase; ANOSIM, Analysis of similarity; ANOVA, Analysis of variance; ASV, Amplicon sequence variant; BCA, Bicinchoninic acid; BMC, Bone mineral content; BMD, Bone mineral density; BV, Blood vessel; BW, Body weight; Ca, Calcium; cDNA, Complementary DNA; COL2A1, Collagen type II alpha 1 chain; CON, Control group; EDTA, Ethylenediaminetetraacetic acid; FCR, Feed conversion ratio; FI, Feed intake; GAPDH, Glyceraldehyde-3-phosphate dehydrogenase; GC-FID, Gas chromatography-flame ionization detection; GP, Growth plate; GSH-Px, Glutathione peroxidase; H&E, Hematoxylin and eosin; HO-1, Heme oxygenase-1; HZ, Hypertrophic zone; MAPK, Mitogen-activated protein kinase; MDA, Malondialdehyde; MMP, Mitochondrial membrane potential; MRCC I, Mitochondrial respiratory chain complex I; MRCC III, Mitochondrial respiratory chain complex III; MZ, Maturation zone; NCBI, National Center for Biotechnology Information; NF-κB, Nuclear factor kappa-B; NRC, National Research Council; Nrf2, Nuclear factor erythroid 2-related factor 2; O_2_^•−^, Superoxide anion; OPG, Osteoprotegerin; P, Phosphorus; PCoA, Principal coordinate analysis; PZ, Proliferative zone; RANK, Receptor activator of nuclear factor kappa-B; RANKL, Receptor activator of nuclear factor kappa-B ligand; ROS, Reactive oxygen species; rRNA, Ribosomal RNA; RT-qPCR, Real-time quantitative polymerase chain reaction; RUNX2, Runt-related transcription factor 2; SCFA, Short-chain fatty acid; SD, Standard deviation; SEM, Standard error of the mean; SOD, Superoxide dismutase; SRA, Sequence Read Archive; T-AOC, Total antioxidant capacity; TD, Tibial dyschondroplasia; TDL, Tibial dyschondroplasia lesion; TSB, Sodium butyrate-supplemented group.
