# Supplementary material for: Dietary Sodium Butyrate Alleviates Thiram-Induced Tibial Dyschondroplasia in Broilers by Improving Redox Homeostasis and Upregulating the Nrf2/HO-1 Pathway
Source: Antioxidants (Basel). 2026 Oct 9;15(10):1302. doi: 10.3390/antiox15101302 (PMC13701647; doi:10.3390/antiox15101302)
Supplement: Supplementary file 1 [file antioxidants-15-01302-s001.zip › antioxidants-4508052-supplementary.pdf]

Table S1. Ingredient and nutrient composition of the basal diet used in this study.

Items	Starter (1–21 days), g/kg
Corn	552.7
Soybean meal, 46% CP	309.6
Corn gluten meal, 60% CP	34.9
Soybean oil	33.0
L-Lysine	2.5
DL-Methionine	3.4
Limestone	12.6
$CaHPO_4$	19.3
NaCl	3.0
Choline chloride	2.0
Vitamin premix[1]	0.3
Mineral premix[1]	1.0
Zeolite powder	1.7
Titanium dioxide[2]	24.0
Total	1000
Calculated nutrient concentrations[3]	
Metabolic energy (MJ/kg)	12.45
Crude protein (g/kg)	217.8
Calcium (g/kg)	10.1
Available phosphorus (g/kg)	4.5
Total phosphorus (g/kg)	6.9
Lysine (g/kg)	11.4
Methionine (g/kg)	4.9
Methionine + cysteine (g/kg)	8.3
Threonine (g/kg)	7.7

[1] The vitamin and mineral premix provided the following per kilogram of diet: vitamin A, 10,000 IU; vitamin D_3, 2000 IU; vitamin E, 10 IU; vitamin K_3, 2.5 mg; vitamin B_1, 1 mg; vitamin B_2, 6 mg; vitamin B_3, 10 mg; vitamin B_5, 40 mg; vitamin B_6, 3 mg; vitamin B_{11}, 0.3 mg; vitamin B_{12}, 0.01 mg; biotin, 0.12 mg; Cu (as copper sulfate), 8 mg; Fe (as ferrous sulfate), 80 mg; Mn (as manganese sulfate), 60 mg; Zn (as zinc sulfate), 40 mg; Se (as sodium selenite), 0.15 mg; I (as potassium iodide), 0.35 mg.

[2] Titanium dioxide was prepared by mixing 4 g titanium dioxide with 20 g ground corn and was included as an indigestible marker.

[3] Calculated nutrient concentrations.

Table S2. Primers used for the RT-qPCR.

Gene	Accession Number	Primer Sequences 5′→3′
Nrf2	NM_001030756.1	F: CTGCTAGTGGATGGCGAGAC<br>R: CTCCGAGTTCTCCCCGAAAG
HO-1	NM_205344.2	F: AAACTTCGCAGCCACACAAC<br>R: GACCAGCTTGAACTCGTGGA
OPG	NM_001033641.2	F: CTGCACGCTTGTGCTCTTGG<br>R: GATGTCCCCGGGTCGTAATG
RANKL	NM_001083361.2	F: ACAAACAGGGCAGGGCAGAAAC<br>R: TTTGCTGTCTCAGGGTCAGAATGC
COL2A1	NM_204426.1	F: ACCTGCCGCGACATCAA<br>R: GTCAATCCAGTAATCTCCGCTCTT
RUNX2	AF445419	F: TAAAGGTGACGGTGGATGG<br>R: TGTGGATTAAAAGGACTTGGTG
GAPDH	NM_204305.1	F: GCCCAGAACATCATCCCA<br>R: CGGCAGGTCAGGTCAACA

The table lists the target genes, corresponding GenBank accession numbers, and primer sequences (5′→3′) used for quantitative real-time PCR. F and R represent the forward and reverse primers, respectively. GAPDH was used as the internal reference gene.
